# Distribution and characterisation of Glucagon-like peptide-1 receptor expressing cells in the mouse brain

**DOI:** 10.1016/j.molmet.2015.07.008

**Published:** 2015-08-05

**Authors:** Simon C. Cork, James E. Richards, Marie K. Holt, Fiona M. Gribble, Frank Reimann, Stefan Trapp

**Affiliations:** 1 Centre for Cardiovascular and Metabolic Neuroscience, Department of Neuroscience, Physiology & Pharmacology, University College London, London, WC1E 6BT, UK; 2 Cambridge Institute of Metabolic Science & MRC Metabolic Diseases Unit, University of Cambridge, Addenbrooke's Hospital, Hills Road, Cambridge, CB2 0QQ, UK

**Keywords:** Glucagon-like peptide-1 receptor, Electrophysiology, Channelrhodopsin, Preproglucagon, GLP-1, PPG, AP, area postrema, BNST, bed nucleus stria terminalis, DMH, dorsomedial nucleus of the hypothalamus, DMV, dorsal motor nucleus of the vagus, Ex-4, Exendin-4, GFAP, glial fibrillary acidic protein, GFP, green fluorescent protein, GLP-1, Glucagon-like peptide-1, GLP-1R, Glucagon-like peptide-1 receptor, NAc, nucleus accumbens, NTS, nucleus of the solitary tract, PARV, parvalbumin, PPG, preproglucagon, PVN, paraventricular nucleus of the hypothalamus, TH, tyrosine hydroxylase, VTA, ventral tegmental area, YFP, yellow fluorescent protein

## Abstract

**Objective:**

Although Glucagon-like peptide 1 is a key regulator of energy metabolism and food intake, the precise location of GLP-1 receptors and the physiological relevance of certain populations is debatable. This study investigated the novel GLP-1R-Cre mouse as a functional tool to address this question.

**Methods:**

Mice expressing Cre-recombinase under the *Glp1r* promoter were crossed with either a ROSA26 eYFP or tdRFP reporter strain to identify GLP-1R expressing cells. Patch-clamp recordings were performed on tdRFP-positive neurons in acute coronal brain slices from adult mice and selective targeting of GLP-1R cells *in vivo* was achieved using viral gene delivery.

**Results:**

Large numbers of eYFP or tdRFP immunoreactive cells were found in the circumventricular organs, amygdala, hypothalamic nuclei and the ventrolateral medulla. Smaller numbers were observed in the nucleus of the solitary tract and the thalamic paraventricular nucleus. However, tdRFP positive neurons were also found in areas without preproglucagon-neuronal projections like hippocampus and cortex. GLP-1R cells were not immunoreactive for GFAP or parvalbumin although some were catecholaminergic. GLP-1R expression was confirmed in whole-cell recordings from BNST, hippocampus and PVN, where 100 nM GLP-1 elicited a reversible inward current or depolarisation. Additionally, a unilateral stereotaxic injection of a cre-dependent AAV into the PVN demonstrated that tdRFP-positive cells express cre-recombinase facilitating virally-mediated eYFP expression.

**Conclusions:**

This study is a comprehensive description and phenotypic analysis of GLP-1R expression in the mouse CNS. We demonstrate the power of combining the GLP-1R-CRE mouse with a virus to generate a selective molecular handle enabling future *in vivo* investigation as to their physiological importance.

## Highlights

• This transgenic mouse allows accurate evaluation of the distribution of GLP-1 receptor expressing cells.
• GLP-1 depolarises PVN, BNST and hippocampus neurons.
• GLP-1R expressing cells can be manipulated *in vivo* using this transgenic mouse.

## 1 Introduction

Glucagon-like peptide 1 (GLP-1) is traditionally recognised as a peripheral incretin hormone. Released postprandially from intestinal L-cells, it binds to GLP-1 receptors (GLP-1R) on pancreatic β-cells to increase insulin secretion. Additionally, GLP-1 acts as a neuropeptide and is produced by preproglucagon (PPG) neurons located in the lower brainstem, primarily in the caudal nucleus tractus solitarii (NTS) and the intermediate reticular nucleus [1,2].

Numerous rodent studies have addressed the question of GLP-1's action within the brain (for review see: [3,4]). Most studies have examined the effects of cerebroventricular injection of GLP-1 or its stable analogue exendin-4 (Ex-4) on food intake [5], but other reported effects include changes in heart rate and blood pressure, as well as effects on learning, memory and thermogenesis [6–9]. Furthermore, central GLP-1 appears to be involved in mediating the rewarding effect of food. Activation of GLP-1R within the ventral tegmental area (VTA) and nucleus accumbens (NAc) results in a significant decrease in the consumption of high calorie food and reduced body weight gain over 24 h [10].

Although the exact mechanism by which GLP-1 affects the brain is unclear, it has been demonstrated that within the VTA and NAc, GLP-1 appears to bind to presynaptic GLP-1R on glutamatergic terminals facilitating glutamate release, without triggering direct postsynaptic effects [11,12].

It has previously been shown that GLP-1 producing neurons in the NTS send ascending axons to forebrain and brainstem regions associated with metabolic and autonomic control in the mouse, including the arcuate nucleus (ARC), hypothalamic paraventricular nucleus (PVN), rostral ventrolateral medulla and dorsomedial hypothalamus (DMH) [1,13]. These projections correlate well with the pattern of GLP-1R expression throughout the brain of the rat [14].

Given the potent effects of central GLP-1 on food intake, it is important to fully elucidate the exact expression profile of GLP-1R throughout the brain. The distribution of GLP-1R mRNA has previously been assessed in the rat brain by *in situ* hybridisation [14]; however, little is known about the distribution in mouse. This gap in knowledge is notable, especially given the increased usage of transgenic mouse models in metabolic physiology.

Here we detail the distribution of GLP-1R expressing cells throughout the mouse brain using a novel transgenic model, in which cre-recombinase is expressed under the control of the *Glp1r* gene. We show that GLP-1R expression correlates well with that observed in the rat [14], non-human primates [15] and with the projection pattern of mouse PPG neurons [1,13]. We also demonstrate that GLP-1R expressing cells do not exhibit immunoreactivity for glial fibrillary acid protein (GFAP) or parvalbumin (PARV), but do show tyrosine hydroxylase (TH) immunoreactivity in some areas. Furthermore, we establish that this mouse model is amenable to manipulation of specific subsets of GLP-1R expressing cells with cre-dependent viral gene transfer. Finally, we assess the feasibility of using this mouse model for *in vitro* analysis using brain slice patch-clamp recordings by recording electrical responses to exogenous GLP-1 from GLP-1R expressing cells in several brain regions.

## 2 Material and methods

### 2.1 Animals

All animal experimentation was carried out in accordance with the UK Animals (Scientific Procedures) Act, 1986, and had the required ethical approval. The entire study was performed on GLP-1R-CRE mice that express cre-recombinase under the *Glp1r* promoter. Generation of these mice has been described in detail before [16]. These mice were crossed with ROSA26-tdRFP or ROSA26-eYFP reporter strains [17] to enable fluorescent detection of cells expressing GLP-1R by cytosolic tdRFP or eYFP expression, respectively. A total of 3 GLP-1R-CRE-eYFP and 8 GLP-1R-CRE-tdRFP mice of both sexes were used for the immunofluorescence study, whereas the electrophysiological study was performed entirely with the GLP-1R-CRE-tdRFP strain. Mice were kept on a 12 h light – 12 h dark cycle, had access to food and water *ad libitum*, and were 8–16 weeks old (weighing between 25 and 35 g) when used for experiments. Females were consistently lighter than males.

### 2.2 Immunofluorescence

Mice were anaesthetised with urethane (1.5 g/kg; i.p.) and perfuse-fixed transcardially with heparinised 0.1M phosphate buffer (PB) followed by 4% paraformaldehyde in 0.1M PB, pH 7.4. Brains were removed from skulls, post-fixed overnight in the same fixative before being cryoprotected in 30% sucrose in 0.1M PB. Brains were sectioned coronally at a thickness of 30 μm (1:5 series) from the olfactory bulbs to the spinomedullary junction.

#### 2.2.1 Immunohistochemical identification of eYFP or tdRFP

Tissues were blocked with 0.1% Triton X-100 and 10% normal goat serum diluted in 0.1M PB for 1 h at room temperature. GLP-1R-expressing cells were detected with antibodies raised against either green fluorescent protein (GFP; Catalogue #AB13970, lot #623923; Abcam, Cambridge MA, USA) or dsRED (Clontech #632496). These were added to the blocking solution in a 1:1000 dilution and incubated overnight at 4 °C. Subsequently, sections were washed 3 times for 5 min in 0.1M PB at room temperature, followed by incubation with a fluorescently labelled secondary antibody (1:500 dilution) in blocking solution for 2 h. Either an anti-chicken Alexa Fluor 488 (Catalogue# **A-11039**, Invitrogen) or an anti-rabbit Cy3 (Catalogue# C2306, Sigma Aldrich, St Louis MO, USA) was used. Sections were washed again as previously described, then mounted onto polylysine-coated slides (Catalogue# 631-1349, VWR), air dried and coverslipped with Vectashield mounting medium (Catalogue# H-1200, Vector Labs).

#### 2.2.2 Double immunofluorescence

Staining was performed as described above with the exception of using sheep serum rather than goat for TH staining. Sections were then incubated with anti-GFP and anti-TH (1:1000 dilution, catalogue# sc-14007, Santa Cruz) or anti-GFP and anti-GFAP conjugated to Cy3 (catalogue# C9205, Sigma Aldrich, St Louis MO, USA), or anti-dsRED and anti-parvalbumin (1:5000 dilution, catalogue# 235, Swant, Switzerland) overnight at 4 °C. Following primary antibody incubation sections were washed as previously described and incubated with the appropriate secondary antibody: for TH the anti-rabbit conjugated to Cy3, and for parvalbumin the Alexa Fluor 488 goat anti-mouse (catalogue# A10680, Invitrogen), respectively. Sections were then washed and mounted as described above.

#### 2.2.3 Data collection and analysis

Stained sections were analysed on an upright epi-fluorescence microscope (Leica DMRB, Leica Microsystems, Milton Keynes, UK) equipped with a Retiga 3000 colour digital camera (QImaging, Surrey, Canada). Images were captured using QCapture software (QImaging) and subsequently exported to Corel Photo-Paint X3 (Corel Corporation, Ottawa, Canada) where brightness and contrast were adjusted. Microsoft Image Composite Editor (Microsoft Corporation, Redmond, USA) was used to produce montages from several frames.

### 2.3 Viral targeting

Adeno-associated virus (AAV) particles were produced as described by Murray et al. [18]. Briefly, to produce rAAV virions containing a 1:1 ratio of type 1 and type 2 capsid proteins human embryonic kidney (HEK) 293 cells were co-transfected with the AAV plasmid pAAV-EF1a-double floxed-hChR2(H134R)-EYFP-WPRE-HGHpA (Addgene plasmid # 20298) and AAV1 (pH21), AAV2 (pRV1) helper plasmids as well as the adenovirus helper plasmid pFΔ6 using calcium phosphate. 64 h post-transfection, cells were harvested, digested with sodium deoxycholate allowing AAVs to be purified from lysates using 1 ml HiTrap heparin columns (Sigma). Eluted virions were concentrated using Amicon Ultra-4 centrifugal filter devices (Millipore U FC810024).

Adult GLP-1R-CRE–tdRFP mice were anaesthetised with ketamine (75 mg/kg; i.m.) and medetomidine (0.5 mg/kg; i.m.). Deep anaesthesia was confirmed by the absence of a pedal withdrawal reflex to a toe pinch. The head was fixed in a stereotaxic frame and the skull was exposed to allow a cranial window to be drilled at the correct location for a unilateral microinjection (150 nl; 50 nl/min) of a solution containing viral particles of EF1a-double floxed-hChR2(H134R)-EYFP AAV serotype 1/2 to target GLP-1R neurons. The coordinates used to target the PVN were 0.82 mm caudal and 0.1 mm lateral from Bregma and 4.75 mm ventral from the surface of the skull at Bregma. After removal of the injection needle the skin was sutured and anaesthesia reversed with atipamezole (1 mg/kg; i.m.). Post-operative analgesia (Buprenorphine, 0.05 mg/kg per day, s.c) was given for two days. Viruses were incubated for 3 weeks before injected animals were transcardially perfused-fixed as detailed above.

### 2.4 Electrophysiology

Coronal (250 μm thick) brain slices were obtained from adult transgenic mice of either sex as described previously [2]. Slices were kept at 34 °C in artificial cerebrospinal fluid (ACSF) of the following composition (in mmol/l): 118 NaCl, 3 KCl, 25 NaHCO_3_, 1 MgCl_2_, 2 CaCl_2_, 10 glucose; pH 7.4. Experimental solutions were constantly bubbled with 95% O_2_/5% CO_2_. Glucagon-like peptide 1 (7–36) amide (#2082) was obtained from R&D systems and prepared as a 100 μM stock solution in MilliQ H_2_O. Whole-cell recording were carried out in ACSF at 28–32 °C using a ‘low Cl’ pipette solution of the following composition (in mM): 120 K-gluconate, 5 HEPES, 5 BAPTA, 1 NaOH, 1 MgCl_2_, 1 CaCl_2_, and 5 K_2_ATP, 0.25 NaGTP; pH 7.2. The junction potential of −10 mV for this pipette solution was added offline. All figures and values in the results are corrected for the junction potential.

Patch-clamp recordings in the voltage-clamp or current-clamp mode were performed on visually identified red fluorescent cells within the bed nucleus of the stria terminalis (BNST), the hypothalamic PVN and the hippocampus using an EPC-9 amplifier and Pulse/Pulsefit software (HEKA-Electronics, Germany). In voltage-clamp membrane resistance was determined by 500 ms hyperpolarising voltage pulses of 40 mV amplitude from the holding potential every 20s. Data are given as mean ± one S.E.M. Statistical significance was tested using a paired or unpaired t-test as appropriate, p-values < 0.05 (*) or < 0.01 (**) were considered significant.

## 3 Results

GLP-1R-tdRFP and -eYFP expressing mice showed similar intensity of fluorescent reporter expression throughout the murine brain, with both strains exhibiting good somatic and proximal neurite staining. Distal neurites were only observed in certain brain regions, assumed to be a result of lower cell density. For example, cells in the PVN showed somatic and proximal neurite staining only, whereas cells in the BNST showed both proximal and more distal neurite staining. Both tdRFP- and eYFP-expressing mice were used in this study and both tdRFP- and eYFP-immunoreactive cells will hereby be referred to as GLP-1R cells.

### 3.1 Distribution of GLP-1R cells

**Figure 1:**
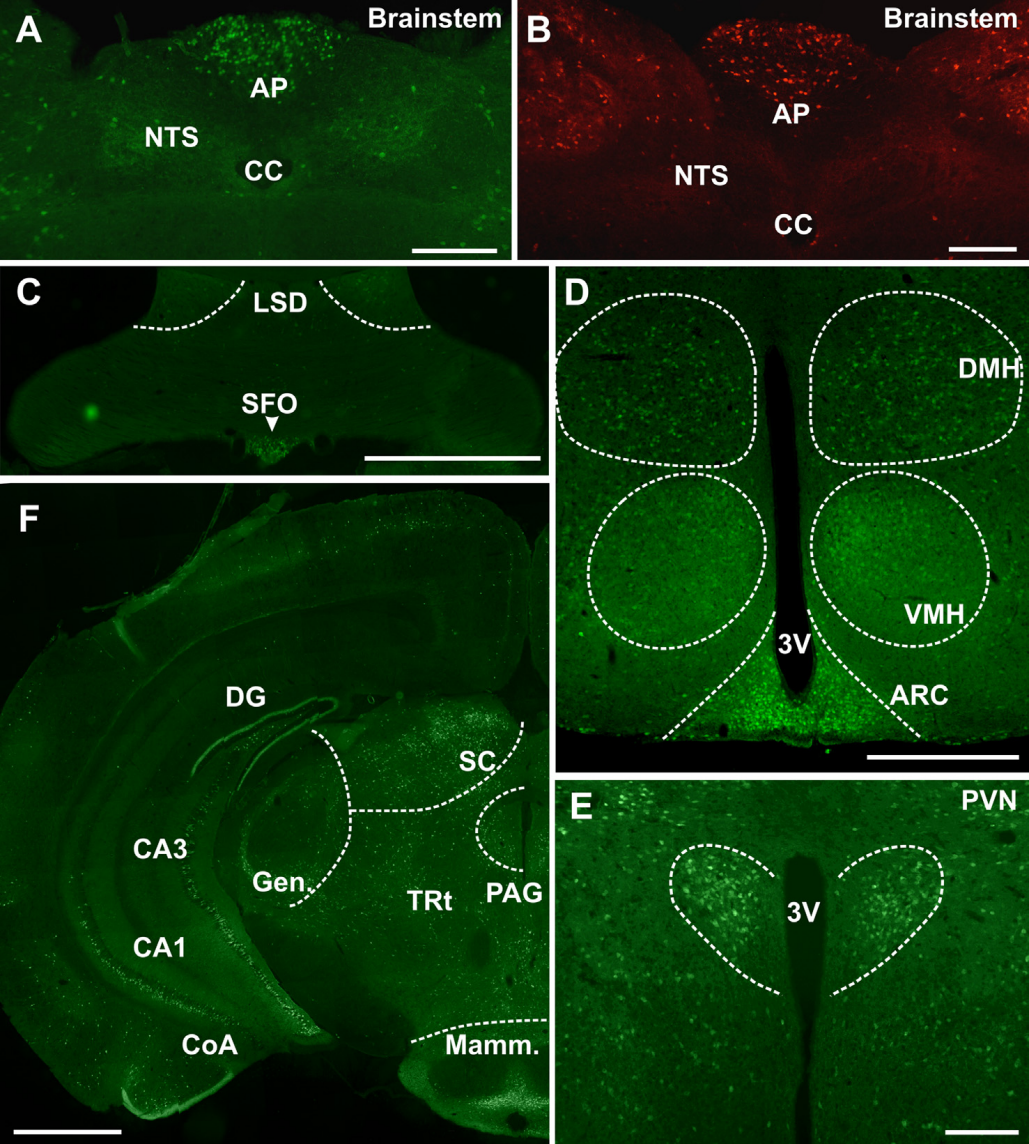
GLP-1 receptor expressing cells in the CNS. **A, B**. Demonstrates the reporter expression in the dorsal vagal complex of the eYFP and tdRFP mice. Expression was equivalent in the area postrema (AP), nucleus tractus solitarius (NTS) and dorsal motor nucleus of the vagus (DMV). High levels of reporter expression were observed in the area postrema (**A**, **B**) dorsal lateral septum, subfornical organ (**C**), ventrolateral hypothalamus, arcuate (**D**), hypothalamic paraventricular nucleus (**E**) superior colliculus and periaqueductal grey (**F**). Expression was also observed, albeit at lower levels, in the nucleus tractus solitarius (**A, B**), dorsomedial hypothalamus (**D**), hippocampus, geniculate, thalamic reticular formation and mammilliary nucleus (**F**). Scale bars: **A**, **B**, **E** = 200 μm. **C**, **F** = 1 mm. D = 500 μm. Abbr: CC: central canal, AP: area postrema, NTS: nucleus tractus solitarius, LSD: dorsal lateral septum, SFO: subfornical organ, DMH: dorsomedial hypothalamus, VMH: ventromedial hypothalamus, ARC: arcuate nucleus, DG: dentate gyrus, Gen: geniculate nucleus, SC: superior colliculus, PAG: periaqueductal grey, CoA: cortical amygdala, TRt: thalamic reticular formation, Mamm: mammillary nucleus, PVN: paraventricular nucleus, 3V: 3rd ventricle.

GLP-1R expressing neurons were examined from the spinomedullary junction to the olfactory bulb of the mouse brain. Densely grouped GLP-1R neurons were observed both in the area postrema (AP; Figure 1A, B) and in the subfornical organ (SFO; Figure 1C), indicating sites where gut-released GLP-1 may directly influence neuronal activity. Interestingly, only few GLP-1R cells were found in the organum vasculosum lamina terminalis, the most rostral circumventricular organ. Many GLP-1R neurons were also found throughout hypothalamic nuclei (Figure 1D–E), particularly in the PVN, DMH and ARC, in-line with *in situ* hybridisation and immunocytochemical studies from rat and primate (Table 1). Furthermore, considerable numbers of GLP-1R neurons were observed in the periaqueductal grey, the mammillary recess, the central nucleus of the amygdala and the caudal hippocampus (Figure 1F).

**Figure 2:**
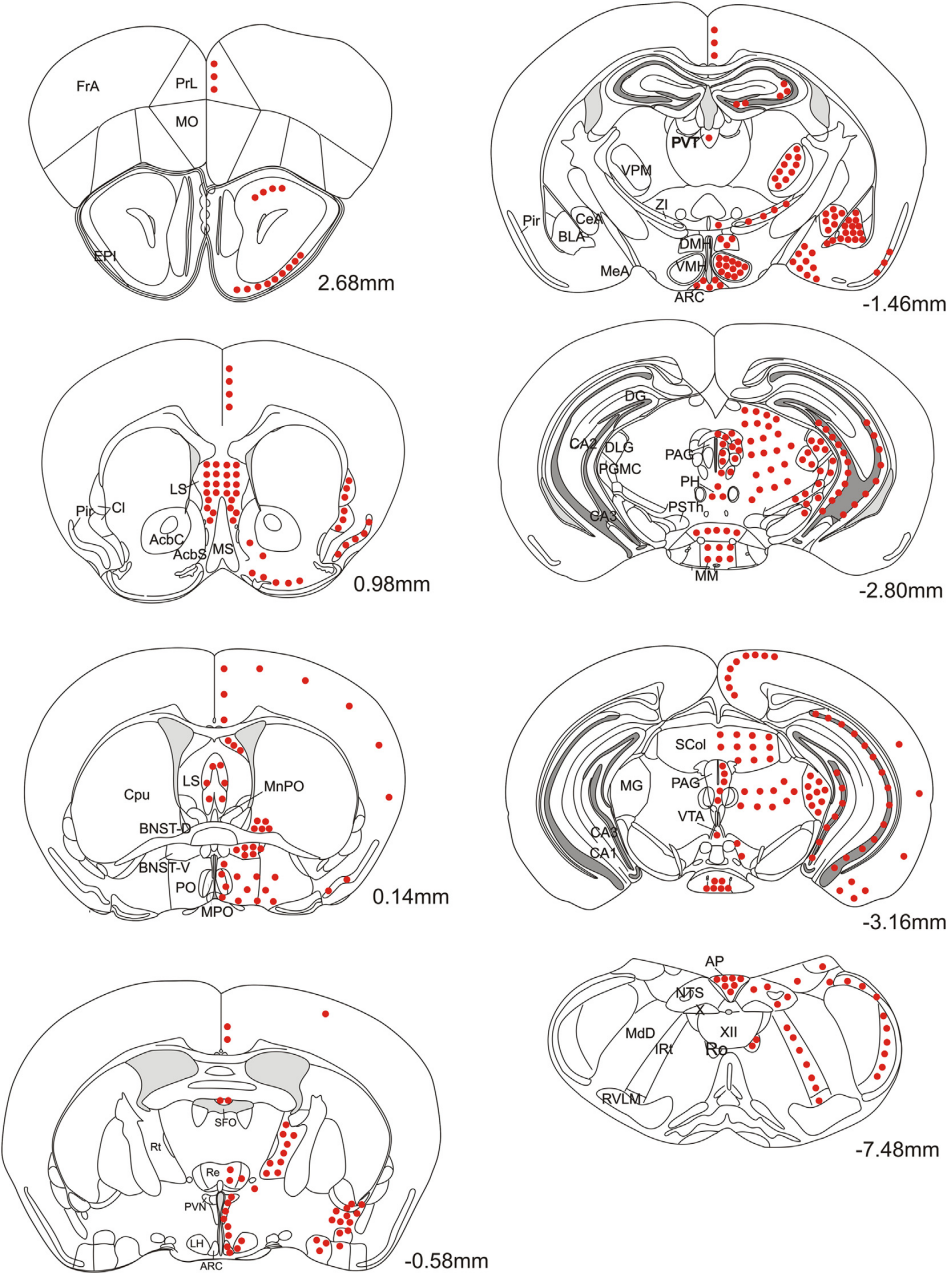
Diagrams of coronal sections showing the distribution of GLP-1R expressing cell bodies in the brains of GLP-1R-Cre mice. Filled circles represent the presence of YFP- or RFP-immunoreactive somata. The density of the filled circles indicates the relative density of the RFP-positive somata in each brain region. Drawings are based on the Paxinos Mouse Brain Atlas and numerical values next to each section indicate the rostro-caudal position in relation to Bregma. Abbreviations: PrL, prelimbic cortex; FrA, frontal association cortex; MO, medial orbital cortex; EPI, external plexiform layer; LS, lateral septum; AcbC, nucleus accumbens core; AcbS, nucleus accumbens shell; MS, medial septum nucleus; Cl, claustrum; Pir, piriform cortex; Cpu, caudate putamen; BNST-D/V, bed nucleus of the stria terminalis dorsal/ventral; PO, preoptic area; MPO, medial preoptic area; MnPO, median preoptic area; SFO, subfornical organ; Re, reuniens thalamic nucleus; PVN, paraventricular nucleus; LH, lateral hypothalamus; ARC, arcuate nucleus; Rt, reticular nucleus; PVT, thalamic paraventricular nucleus; VPM, ventral posteromedial thalamic nucleus; CeA, central amygdala; BLA, basolateral amygdala; DMH, dorsomedial hypothalamus; VMH, ventromedial hypothalamus; PAG, periaqueductal grey area; PH, posterior hypothalamus; PSTh, parasubthalamic nucleus; MM, mammillary nucleus; DG, dentate gyrus; DLG, dorsolateral geniculate nucleus; PGMC, pregeniculate nucleus magnocellular part; SCol, superior colliculus; VTA, ventral tegmental area; MG, medial geniculate nucleus; AP, area postrema; NTS, nucleus tractus solitarius; XII, hypoglossal nucleus; Ro, nucleus of roller; IRt, intermediate reticular nucleus; MdD, dorsal medullary reticular nucleus; RVLM, rostral ventrolateral medulla.

A schematic representation of the distribution of GLP-1R neurons throughout the mouse brain is given in Figure 2 and the density of GLP-1R neurons in individual brain nuclei is quantified in Table 1. Within the lower brainstem, GLP-1R neurons were scattered towards the lateral aspects of the NTS and throughout the intermediate reticular nucleus. Scattered neurons were also observed in the rostral ventrolateral medulla, locus coeruleus and nucleus of Roller. Further rostral few neurons were observed in the geniculate nucleus, mammillary nucleus, pretectal nucleus, and superior colliculus.

**Figure 3:**
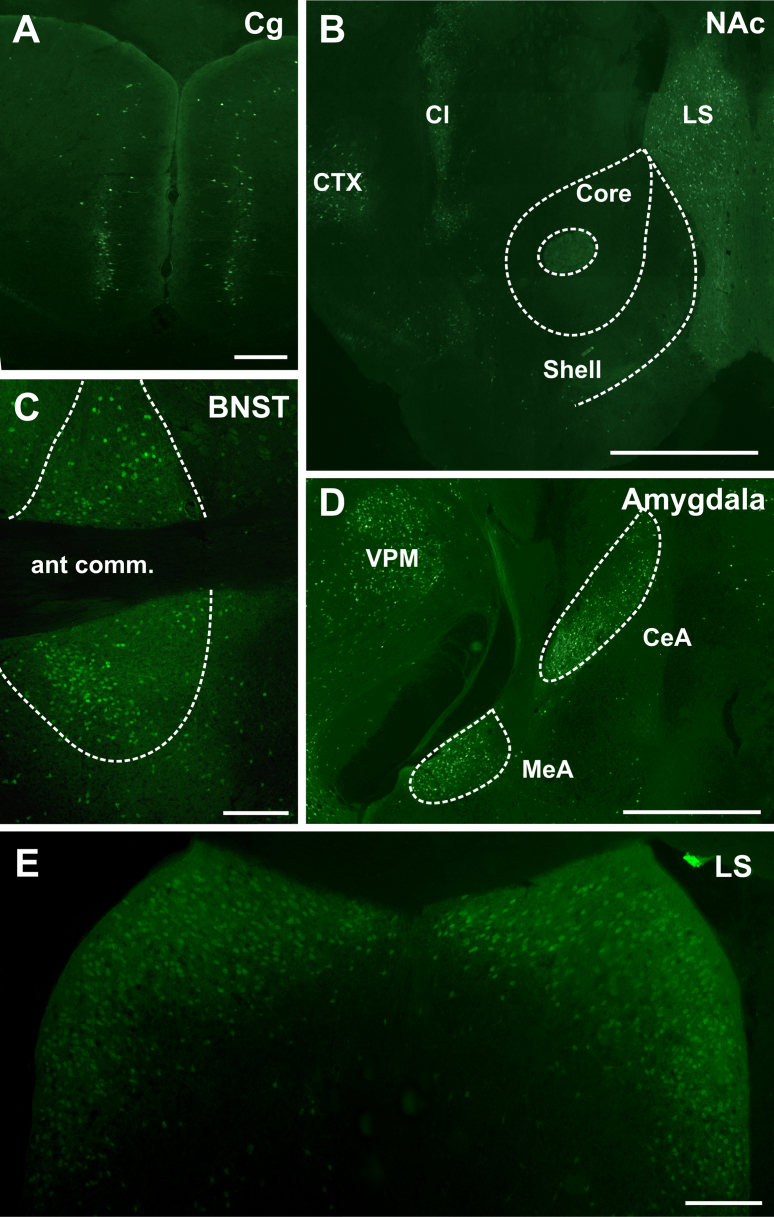
GLP-1 receptors in the meso-limbic system. Photomicrographs of YFP-immunoreactive cells in (**A**) cingulate cortex, (**B**) the nucleus accumbens, lateral septum, claustrum and cortex, (**C**) the bed nucleus of the stria terminals, (**D**) amygdala and ventral posteromedial thalamic nuclei, and (**E**) the dorsal lateral septum. Abbr: Cl: claustrum, CTX: cortex, ant comm: anterior commissure, CeA: central amygdala, LS: lateral septum, MeA: medial amygdala, VPM: ventral posteromedial thalamic nuclei. Scale bars: **A**, **C**, **E** = 200 μm; **D** = 300 μm; **B** = 1 mm.

In the diencephalon, GLP-1R neurons were located at a high density throughout the hypothalamus, particularly in the magnocellular and parvocellular subdivisions of the PVN, DMH and ARC, whereas smaller numbers of neurons were scattered throughout the lateral and posterior hypothalamus. In the thalamus, GLP-1R neurons were observed in the ventral thalamic nuclei, the paraventricular nucleus, the zona incerta, the nucleus reuniens, parasubthalamic nucleus, reticular thalamic nucleus, posterior thalamus, parafascicular thalamus and precommissural area. In the hippocampus, few neurons were observed in the rostral aspect of the CA3 region and the dentate gyrus. Many more neurons, however, were observed in the caudal CA3 and CA1 regions where the hippocampus extends ventrally (Figure 1F). In the telencephalon, expression was observed in the plexiform cell layer of the olfactory bulb, posterior and ventral subnuclei of the BNST (Figures 1C and 3C), lateral septum (Figure 3E, ventral tenia tecta, claustrum (Figure 3B), preoptic area, SFO, central, medial and basolateral amygdaloid nuclei (Figure 3D). Few scattered neurons were located in both the nucleus accumbens shell and core (Figure 3B).

Within the neocortex two regions were observed that showed populations of GLP-1R neurons, the piriform and cingulate cortices (Figures 2, 3A, 5B, C). The remainder of the cortex was virtually devoid of GLP-1R neurons with very occasional rare scattered neurons found, as indicated in Figure 2.

**Figure 4:**
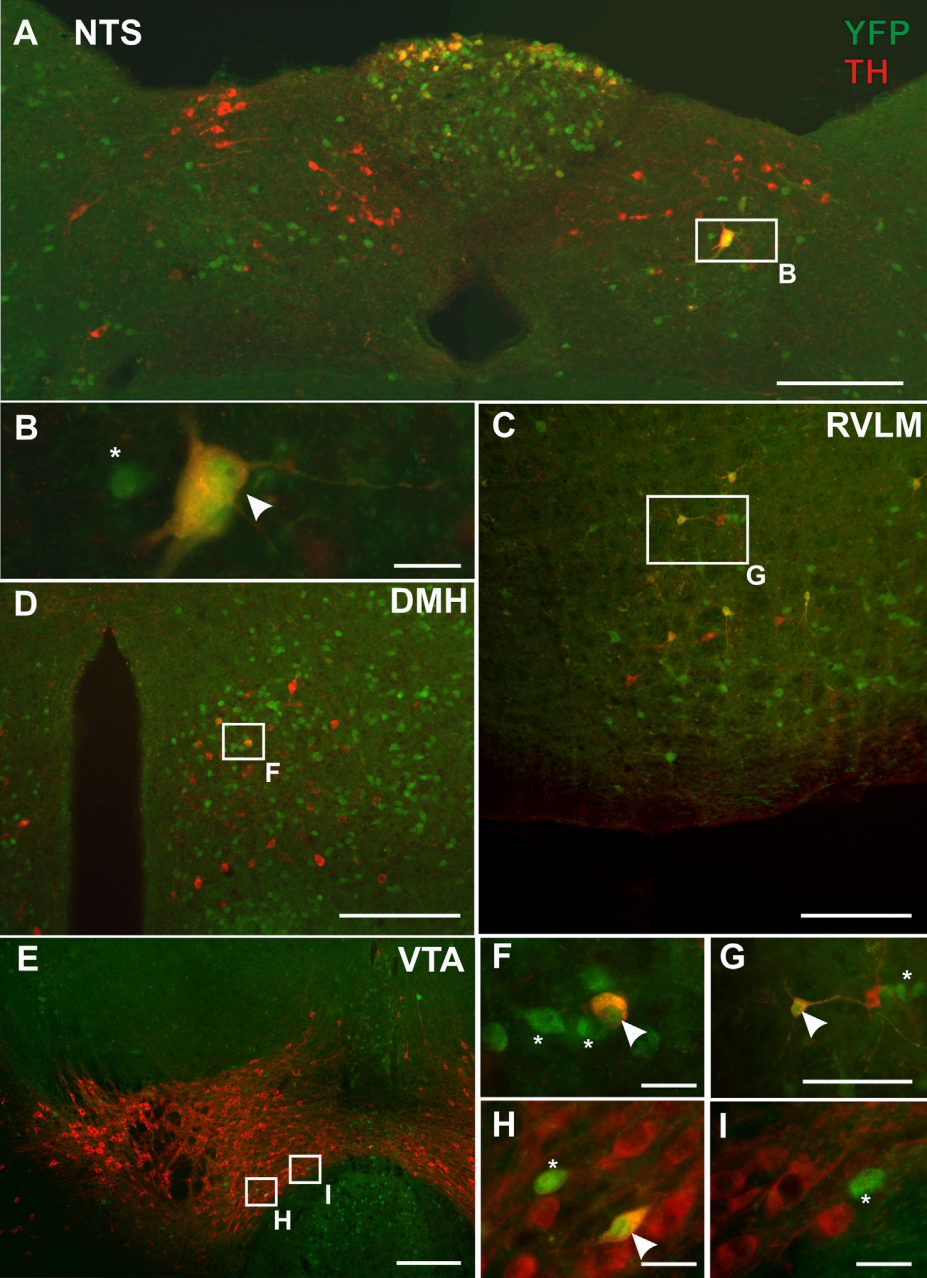
Dual immunofluorescence of GLP-1R-eYFP expressing neurons and tyrosine hydroxylase (TH) expressing neurons throughout the brain. **A**. Only few TH neurons in the NTS were found to co-localise with eYFP expressing neurons. Conversely, almost all TH positive neurons in the AP co-expressed GLP-1R. **B**. Higher magnification image of boxed region in **A**. **C**. Around half of all TH positive neurons in the RVLM co-expressed GLP-1R, however the majority of GLP-1R neurons were TH-negative. **D**. A few TH positive neurons in the DMH were found to have eYFP staining, however the majority did not. **E**. Only a few TH positive neurons in the VTA expressed GLP-1R. Indeed the VTA contained very few GLP-1R positive somata. **F–I**. Scale bars in A, C, D and E = 200 μm. B, F, H and I = 20 μm. G = 50 μm. Arrow heads in B, F–I represent dual labelled neurons. * = YFP positive, TH negative neurons.

The mesolimbic system exhibited high densities of GLP-1R neurons in the BNST (Figure 3C), the amygdala (Figure 3D) and the lateral septum (LS; Figure 3E). Interestingly, only few somata were identified in the ventral tegmental area (VTA) (Figure 4E) and NAc (Figure 3B), although both areas have previously been investigated in relation to the effects of GLP-1R activation on food intake in rats.

### 3.2 Co-localisation with catecholamine neurons

Catecholamine neurons were revealed through immunofluorescent labelling of tyrosine hydroxylase (TH) on brains of GLP-1R-eYFP mice. In the medulla, TH-immunoreactive neurons were identified throughout the AP, NTS and ventrolateral medulla (VLM). Within these areas, a subset of TH-immunoreactive neurons co-localised with eYFP expression (Figure 4A–C). Within the AP the majority of TH-immunoreactive neurons were localised at the dorsal surface, and in agreement with previously published work demonstrating GLP-1 receptors on these cells [19], almost all TH neurons co-expressed eYFP. Within the NTS, TH-immunoreactive neurons were most commonly located towards the midline, whereas eYFP expressing cells were more commonly expressed towards the lateral edge, however, there were a few instances where colocalisation occurred (Figure 4 A, B).

TH-immunoreactive neurons were found scattered throughout the VLM, and around half were found to co-localise with eYFP positive neurons (Figure 4C, G). However the number of eYFP positive neurons observed was much greater when compared with TH-immunoreactive neurons. Altogether, only a small proportion of GLP-1R neurons in the VLM were found to be TH-immunoreactive.

The midbrain VTA has a high density of TH-immunoreactive neurons throughout, but a low level of eYFP-positive somata (Figure 4E, H, I). Of these eYFP-positive neurons, a small number, predominantly located on the ventral border, were found to contain TH-immunoreactivity.

Within the hypothalamus, TH-immunoreactive neurons were found scattered throughout the dorsomedial hypothalamus (DMH), a small proportion of these were found to co-stain for eYFP (Figure 4D, F). However the majority of GLP-1R neurons in the DMH were not TH-immunoreactive. TH-immunoreactive neurons were also identified in the PVN, with the highest densities located in the caudal PVN. Here, no eYFP neurons were identified as being TH immunoreactive, however eYFP neurons were interspersed amongst TH-immunoreactive neurons.

### 3.3 Co-localisation with parvalbumin neurons

**Figure 5:**
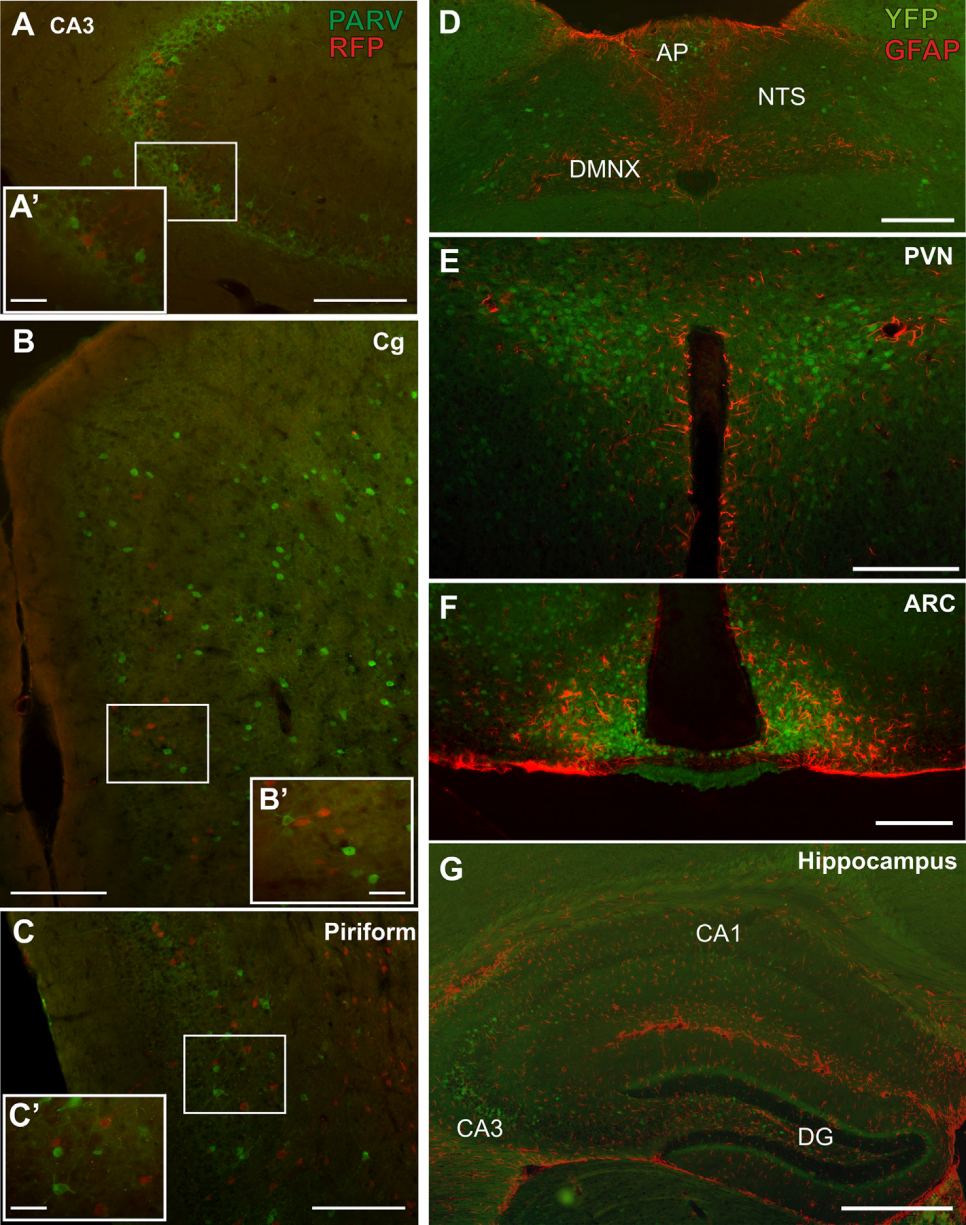
Dual immunofluorescence of GLP-1R-tdRFP expressing neurons and parvalbumin (PARV) expressing neurons (**A–C**) and GLP-1R-eYFP expressing cells and GFAP immunofluorescence (**D–G**). PARV positive neurons were present in the hippocampus (**A**), cingulate (**B**), piriform (**C**) and thalamic regions (not shown). No tdRFP-positive cell was found to co-express PARV in any region, although the populations did intermix. Similarly, GFAP was expressed throughout the CNS, and although intermixed with eYFP expression, no co-expression was identified. Scale bars in A-F = 200 μm; A’–C’ = 50 μm and G = 500 μm.

Parvalbumin (PARV)-immunoreactive neurons were predominately found in the hippocampus, cortex and thalamus (Figure 5A–C). In the thalamus, PARV staining was restricted to the reticular nucleus and superior colliculus. Within these regions, no cells were found to be immunoreactive for both RFP and PARV. Similarly, in the hippocampus, cortex and reticular nucleus, populations of both RFP- and PARV-immunoreactive neurons intermingled, but the two antigens were not found to co-localise.

### 3.4 Co-localisation with GFAP

Astrocytes were detected by immunofluorescent labelling of glial fibrilliary acidic protein (GFAP) in GLP-1R-eYFP brain sections (Figure 5D–G). Within the dorsal vagal complex (DVC), GFAP-immunoreactive cells were observed throughout the dorsal motor nucleus of the vagus (DMNX), the midline of the NTS and along the border of the AP. No co-localisation between eYFP immunofluorescence and GFAP immunofluorescence was observed in the NTS or the DMNX. GFAP staining was predominately observed along the midline of the DVC, whereas eYFP-positive cells were generally observed more laterally. Within the AP, although both GFAP-positive and eYFP-positive cells were found, no co-localisation was observed (Figure 5D).

Similarly in the hypothalamic PVN, GFAP was observed predominately around the border of the 3rd ventricle, with scattered processes observed throughout the PVN. No eYFP-positive cell was observed to contain GFAP immunoreactivity (Figure 5E). The ARC contained relatively large numbers of GFAP positive cells, but, again, there was no evidence of co-expression of eYFP-immunofluorescence and GFAP in individual cells (Figure 5F). A similar observation was also made in the rostral CA3 region of the hippocampus, that exhibited a number of GLP-1R cells, but none of these were immunoreactive for GFAP (Figure 5G).

### 3.5 Selective targeting of GLP-1R neurons *in vivo*

**Figure 6:**
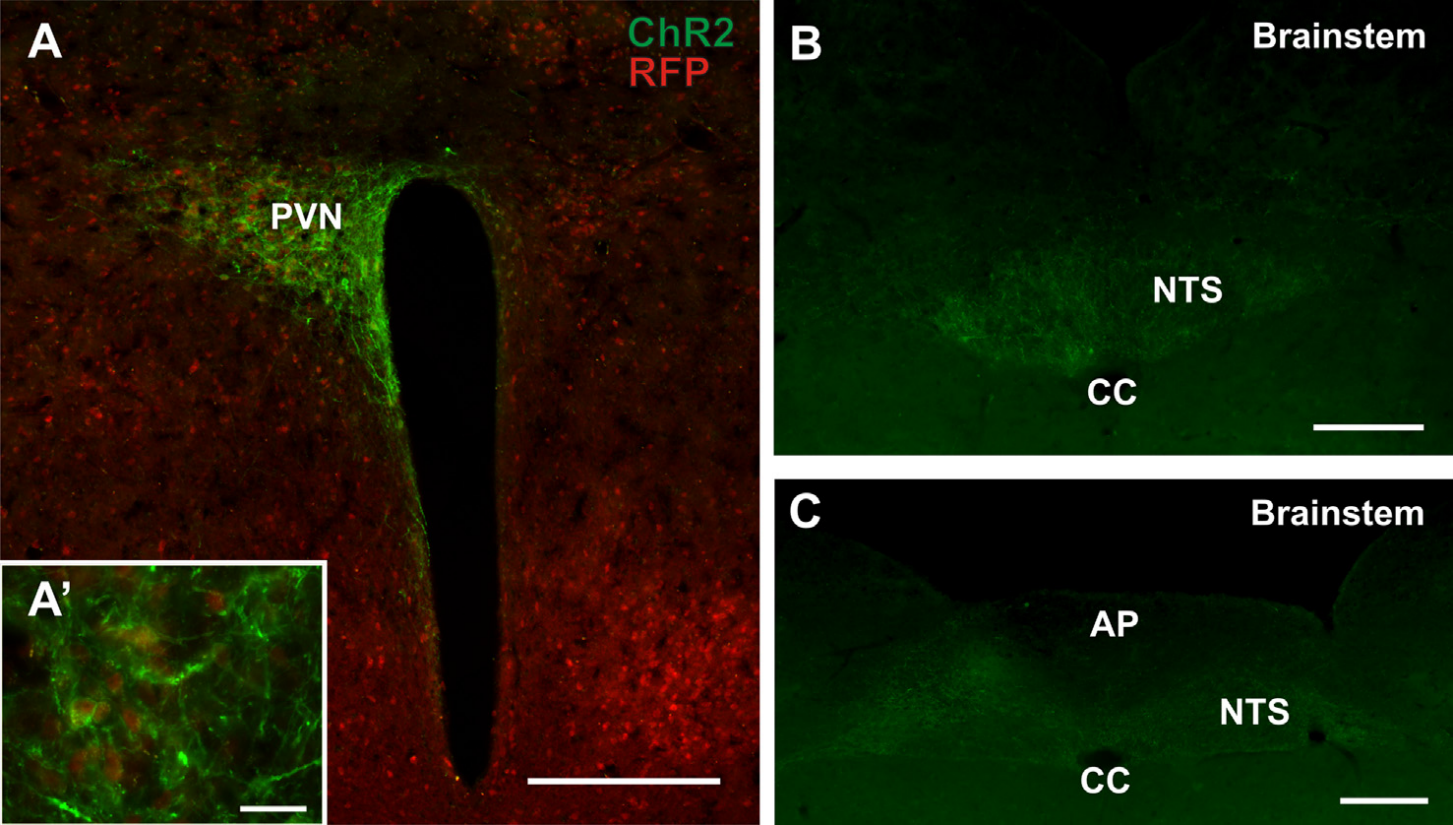
Unilateral microinjection of a cre-dependent AAV expressing ChannelRhodopsin-2 (AAV-flexswitch-ChR2-eYFP) in the hypothalamic PVN. **A**. ChR2 expression (green) is confined to the boundaries of the PVN and expressed only in cells which co-express tdRFP (red). (**A’**) Higher magnification image of ChR2 expressing tdRFP positive neurons in the PVN. This study confirms the presence of active cre-recombinase in these cells and provides proof-of-principle evidence that these mice can be used to virally target GLP-1R expressing cells in discrete nuclei. **B, C**. ChR2-eYFP expressing axons of virally-targeted PVN neurons are distributed primarily in the caudal NTS up to the rostral end of the AP. Scale bar A = 100 μm. A’ = 10 μm. B, C = 200 μm. 3V = 3rd ventricle.

Expression of cre-recombinase in cells where the GLP-1R promoter is active enabled the functional manipulation of GLP-1R neurons in *vivo.* We injected EF1a-double floxed-hChR2(H134R)-EYFP AAV particles into the PVN to express ChR2-eYFP selectively in PVN GLP-1R neurons only (Figure 6A). Axons expressing ChR2-eYFP were primarily found in the caudal NTS (Figure 6B, C) and to a much smaller extent in the VLM (not shown).

### 3.6 Electrical responses of GLP-1R neurons to GLP-1 *in vitro*

In order to verify that tdRFP- or eYFP-immunoreactive neurons did indeed express GLP-1R and that the receptor is functional, whole-cell patch-clamp recordings were performed on tdRFP-neurons in three different brain regions in an *in vitro* brain slice preparation.

#### 3.6.1 PVN

**Figure 7:**
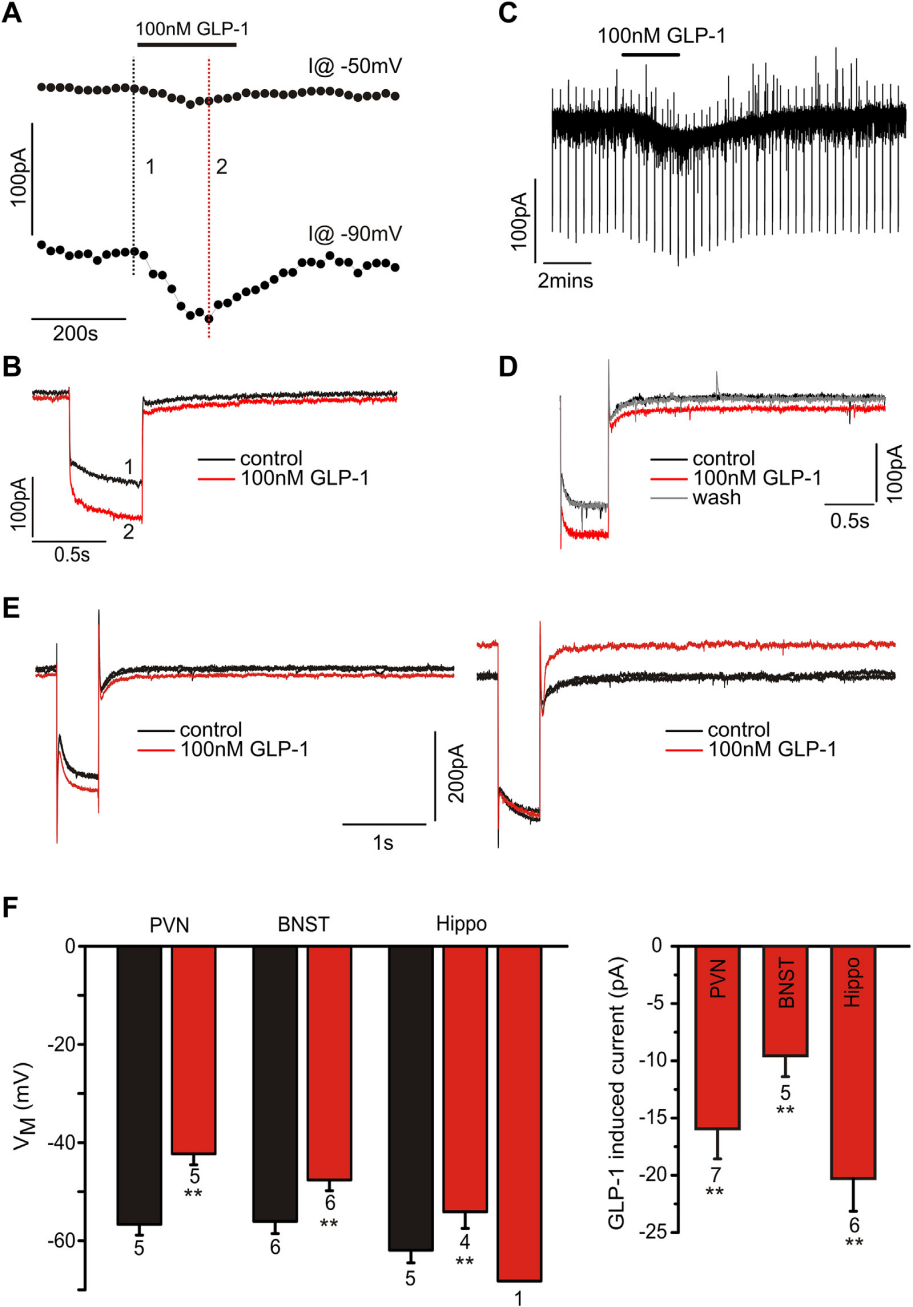
GLP-1 elicits electrical responses in GLP-1R-Cre positive cells in various brain regions. **A, B**. Voltage-clamp recording from an RFP-positive PVN neuron. Bath application of 100 nM GLP-1 elicited a small inward current at a holding potential of −50 mV accompanied by an increase in whole-cell conductance. These effects reversed upon washout of GLP-1 from the bath solution. **C, D**. Similarly, in a BNST neuron bath application of 100 nM GLP-1 elicits an inward current accompanied by an increase in conductance. **E**. Voltage clamp recordings in RFP-positive hippocampal neurons revealed that GLP-1 either elicits an inward current (left panel) as in PVN and BNST, or an outward current. **F.** Mean data from current clamp recordings (left panel) demonstrating that GLP-1 causes a 10–15 mV depolarisation in most neurons tested, and a hyperpolarisation in individual hippocampal cells. Mean data from voltage clamp recordings in the right panel demonstrate the inward current of 10–20 pA amplitude elicited by 100 nM GLP-1 underlying the depolarisation in current clamp. n-numbers are given below the bars. **: p < 0.01.

Seven red-fluorescent PVN neurons were recorded in voltage clamp at a holding potential of −50 mV, and five cells were recorded in current-clamp mode. The mean resting potential in current-clamp mode was −57 ± 2 mV (n = 5). Bath-application of 100 nM GLP-1 caused a depolarisation by 14 ± 1 mV in all five cells that reversed upon washout of the drug. In voltage clamp GLP-1 application induced a reversible inward current of 16 ± 3 pA (n = 7) that was accompanied by a decrease in membrane resistance from 1.1 ± 0.2 GΩ to 0.8 ± 0.1 GΩ (Figure 7A–C, F).

#### 3.6.2 BNST

Six BNST neurons were recorded in current clamp. Their resting potential was −56 ± 3 mV and bath application of 100 nM GLP-1 elicited a depolarisation by 8 ± 1 mV that was reversible upon washout of the drug. An additional five recordings were performed in voltage clamp at a holding potential of −50 mV. In these cells GLP-1 caused an inward current of 10 ± 2 pA (Figure 7D, F).

#### 3.6.3 Hippocampus

Finally, five hippocampal pyramidal neurons were recorded in current clamp and eight cells were recorded in voltage clamp held at −50 mV. The mean resting potential in current-clamp mode was −62 ± 3 mV (n = 5). Bath-application of 100 nM GLP-1 caused a depolarisation by 8 ± 0.3 mV in four of these cells and a hyperpolarisation of 6 mV in the remaining cell. Similarly, in voltage clamp six out of eight cells tested developed a reversible inward current of 20 ± 3 pA under 100 nM GLP-1 (Figure 7E, F), whereas the remaining two cells exhibited an outward current of 63 and 36 pA, respectively.

## 4 Discussion

This study has described the anatomical distribution of tdRFP- or eYFP-immunoreactive cells throughout the brain, from the spinomedullary junction through to the prefrontal cortex of the GLP-1R-Cre mouse. This mouse uses the *Glp1r* promoter to drive expression of the enzyme cre-recombinase, which in turn allows the expression of tdRFP or eYFP in the ROSA26 locus. These fluorescent markers were used to visually identify cells, either directly in acute brain slices used for electrophysiological recordings, or by immunofluorescence in fixed tissue sections. The extent of cellular labelling was comparable between the tdRFP and eYFP strains. In both cases, mostly somata and proximal dendrites were visualised and immunoreactivity did not reveal more distal dendrites or axons. In contrast to our earlier studies on PPG neurons [1,13,20], axon projections of GLP-1R neurons could not be visualised, due to insufficient amounts of RFP or eYFP in these structures. However, targeting the GLP-1R with AAV constructs as discussed below led to sufficient expression of the virally-encoded fluorescent marker in the axons of these cells to allow visualisation of projection targets.

Given that cre-recombinase is expressed under the *Glp1r* promoter, tdRFP or eYFP should identify cells that express the GLP-1 receptor. Clear proof of whether this is indeed the case would be successful labelling with a GLP-1R antibody. However, the paucity of reliable GLP-1R antibodies is well established [21,22], and, consequently, a functional approach was taken in this study. We screened tdRFP-positive cells in a few brain regions with established (e.g. PVN), and with questionable (e.g. hippocampus), GLP-1R expression for electrophysiological responses to GLP-1. The results demonstrated that tdRFP fluorescence indeed signified functional responsiveness to GLP-1, and thus most likely the presence of GLP-1R on these cells. Inevitably numbers of cells tested in this manner are low, and we cannot exclude with certainty that some subpopulations of RFP-immunoreactive cells described in this study might reflect either ectopic expression or lineage tracing [16]. However, within these potential constraints, the GLP-1R-Cre/ROSA26-tdRFP/eYFP mouse is an invaluable tool for examining GLP-1 function within the CNS. Firstly, this mouse obviates the need for notoriously unreliable antibodies for the detection of GLP-1R and therefore offers a greater level of reliability as antibodies raised against eYFP and tdRFP are generally less susceptible to non-specific binding (and their specificity can be easily tested in brain tissue from wildtype mice). Secondly, it allows the identification of GLP-1R expressing cells in live slice preparations and thus permits functional characterisation of defined GLP-1R expressing cells at cellular level *in vitro*. Finally, it enables the use of viral gene transfer to manipulate specific sets of GLP-1R expressing cells *in vivo*.

### 4.1 Distribution of GLP-1R throughout the murine brain

GLP-1R expressing cells were observed throughout the rostrocaudal extent of the mouse brain, and generally correlated with the projection targets of brainstem PPG neurons [1,13,23–25] and the expression of GLP-1R described in rat [14], although some differences were observed. Interestingly, the expression pattern is also very similar to that of a non-human primate (Table 1; [15]), which suggests that the GLP-1 system is largely conserved throughout the brain between mammalian species. This might not be surprising, given that the distribution of GLP-1 producing PPG neurons is also quite conserved between species [1,23,26,27]. The largest numbers of GLP-1R cells were observed in regions associated with autonomic and behavioural control of energy balance, such as the PVN, ARC, DMH and central amygdala. Selective targeting of GLP-1R cells in future studies should shed light on current controversies such as whether GLP-1 action in the ARC affects glucose homeostasis or not [28–30]. Many GLP-1R expressing neurons were also observed in the dorsal lateral septum (DLS) and in the BNST. The DLS has recently been linked to effects of GLP-1 on cocaine-induced behaviour [31] and the BNST is a major projection target for PPG neurons in both rat and mouse [1,32]. The AP and SFO, which form part of the brains' circumventricular organs, also contained large numbers of GLP-1R expressing cells. Moderate numbers of GLP-1R expressing cells were observed in the ventrolateral medulla, which like the PVN harbours presympathetic neurons, and thus both regions might contribute to the effects of GLP-1 on cardiovascular control as well as thermogenesis [8,9,33].

Of particular interest were brain areas such as the VTA and NAc, which have recently received attention for their ability to reduce food intake and reward behaviour following GLP-1R activation [10,34,35]. In these regions, there was little GLP-1R expression, in contrast to observations in rat [14], but in agreement with recent *in situ* hybridisation studies in mouse [31]. Indeed, tracing studies in rats have observed that around 30% of NTS PPG neurons project to the VTA and around 40% project to the NAc [25] suggesting that they are sites of GLP-1 release from brainstem PPG neurons. *In vitro* electrophysiological studies in rats have reported increased glutamatergic signalling onto dopaminergic neurons by GLP-1 acting via presynaptic receptors [11,12]. It is therefore possible to speculate that GLP-1Rs in these regions are present on terminals arising from other sites and the somata of these neurons would most likely be located in distal nuclei, thus providing an explanation for the absence of GLP-1R positive cell bodies in the VTA and NAc. Conversely, this discrepancy could be the result of species variation, since most *in vivo* behavioural studies involving the actions of GLP-1 in these brain regions were performed in rats. This notion may be supported by the fact that very few PPG axons can be found in the NAc of mice.

Previous studies have reported the putative existence of GLP-1R on glial cells throughout the CNS [36,37]. Here, using double immunofluorescence for eYFP and the astroglial marker, GFAP, we found no co-localisation throughout the brain. This is in agreement with a recent study that located GLP-1R on microglia, but not astroglia, in rat spinal cord [38]. It should be noted, however, that immunofluorescent identification of GFAP predominately labelled astrocytic processes, whereas eYFP predominately labelled somata. It is therefore possible that a more specific marker for astrocytic somata may reveal the existence of GLP-1R on glial cells. However, transcriptome analysis of astrocytes failed to demonstrate significant expression of GLP-1R [39] (see also: http://www.networkglia.eu/en/transcriptome).

### 4.2 Projections from GLP-1R expressing PVN neurons

Unilateral injections of AAV into the PVN demonstrated that these neurons express cre-recombinase in the adult animal. Cre-recombinase expression is driven by the GLP-1R promoter, indicating that the red fluorescent PVN neurons do express GLP-1R. Indeed electrophysiological recordings from a small number of these neurons confirmed that GLP-1 elicits an inward current and depolarisation in these cells. Activation of the AAV Flex-switch in the injected animals led to the expression of ChR2-eYFP embedded in the membrane of the transduced cells, which enabled analysis of their specific projection patterns. It was noteworthy that a particularly high density of varicose axons from the PVN GLP-1R neurons was observed in the NTS and DMNX, whereas other known projection targets of PVN neurons, such as the VLM received substantially fewer axon terminals. This finding is in line with studies that found a similar distribution of PVN projections in rat [40–42]. It remains to be established if part of this projection constitutes a feedback loop to modulate the activity of PPG neurons in the NTS. Given that in rat most neurons in the medial NTS, which receive input from unidentified PVN neurons have been shown to be responsive to gastric distension [43], like the PPG neurons, this seems a distinct possibility.

### 4.3 GLP-1R in hippocampus

Various studies have reported effects of GLP-1 in hippocampus, both at the cellular and systemic levels [6,44]. A recent study even reported a reduction in food intake upon injection of Ex-4 into the hippocampus [45]. However, in the absence of any clear innervation with GLP-1 containing axons from PPG neurons in rodents or primates [1,23,26], it is difficult to imagine the source of GLP-1 in hippocampus. Nevertheless, whilst non-human primates do not seem to express GLP-1R in hippocampus [15], both *in situ* hybridisation and the current study identify GLP-1R in parts of the hippocampus of rat and mouse, respectively [14]. The study by Merchenthaler and colleagues reported GLP-1R mRNA expressing cells in the ventral hippocampus of rat, particularly in the CA3 region [14]. The current study confirmed this in mouse, but also identified a small, succinct group of pyramidal cells in the rostral CA3. In contrast, GLP-1 binding studies failed to identify the hippocampus as a target [46]; however, since cell numbers in this region were low, it could be below the detection limit. Additionally, here we show electrical responses of individual identified red fluorescent hippocampal neurons to GLP-1. The recordings indicated two types of electrical responses, either the activation of an inward current, presumably a non-selective cation channel, or the activation of a K^+^ current. The former is an excitatory and the latter an inhibitory response. The combination of the fluorescence identification and the electrical responsiveness of these cells makes it highly likely that these hippocampal neurons indeed express GLP-1 receptors. This raises the question of whether these cells would be exposed to GLP-1 *in vivo*, and, if so, where would this GLP-1 originate? It has been proposed that GLP-1 could reach the hippocampus from the cerebrospinal fluid (CSF), and indeed detectable levels of GLP-1 were found in CSF samples [45]. However, whether CSF GLP-1 originates from gut-derived GLP-1 or is released into the CSF by PPG neurons is currently unclear.

### 4.4 Functional consequences

GLP-1Rs are expressed in regions protected by the blood brain barrier (BBB) as well as those outside. This provides two possible mechanisms through which GLP-1 may exert actions within the brain. It is generally believed that the rapid degradation of GLP-1 in the blood by circulating DPP-IV prevents gut secreted GLP-1 from crossing the BBB [47]. Receptors expressed on cells within circumventricular organs provide a mechanism by which circulating GLP-1 may exert actions within the brain. Indeed, it has been shown that GLP-1 responsive catecholaminergic neurons in the AP project to areas associated with autonomic control, including the parabrachial nucleus and RVLM [19]. However, the existence of GLP-1R in regions protected by the BBB suggests that brain-derived GLP-1 is involved in regulating at least some of the responses attributed to GLP-1 action in the brain. This is further supported by the existence of receptors in regions, which receive dense innervation from brainstem PPG neurons [1,13,23–25]. On the other hand, two of the circumventricular organs, the AP and the SFO, express a high density of GLP-1 receptor cells, but show a rather limited amount of innervation from PPG axons [1,13]. This might point to a role for peripheral GLP-1 in these regions, or to a role for CSF GLP-1. Further work will be needed to fully elucidate the relative contribution of brain-derived vs peripherally-derived GLP-1 on the brain GLP-1 system.

Previous *in vitro* electrophysiological work has focussed on the VTA and NAc. It has been suggested that GLP-1Rs exert their effect through a pre-synaptic enhancement of glutamate release in the VTA and NAc [11,12]. In contrast, in the regions investigated in our study, we consistently showed whole-cell currents elicited by GLP-1 in RFP-positive neurons, indicative of post-synaptic GLP-1 receptors, activation of which opens a non-selective cation channel in most cells or a K^+^ channel in some hippocampal neurons. This discrepancy could indicate why few GLP-1R containing cell bodies are observed in the VTA and NAc in our study and that of Harasta et al. [31]. If they are expressed on axons terminals, their cells bodies may be located in distal nuclei. This would explain why microinjection of GLP-1 or Ex-4 into the VTA and the NAc [10,34,35] elicits functional effects, but few GLP-1R-expressing cell bodies are found in these regions. Alternatively, a species-difference between rat and mouse might exist, leaving mouse insensitive to GLP-1 in the VTA and NAc.

### 4.5 Conclusions

In this study, we have demonstrated that this mouse model provides not only the means to describe the distribution of GLP-1R expressing cells throughout the brain facilitating identification and functional characterisation *in vitro*, but also that it is amenable to genetic targeting of GLP-1R cells with cre-dependent viral gene expression. These properties will allow future studies of functional interference with specific subsets of GLP-1R expressing cells *in vivo*, as well as circuit mapping, and development of bespoke mouse strains containing knock outs of desired proteins in GLP-1R expressing cells.

The difficulty of identifying neurons expressing the GLP-1R is made all the more complex by the lack of reliable antibodies. Here we have circumnavigated this problem by using a transgenic mouse, which expresses Cre recombinase under the control of the *Glp1r* promoter. Although this negates the need for antibodies to detect the expression of the GLP-1R, it is noteworthy that the expression of floxed fluorescent proteins requires only *Glp1r* promoter activity at some point during development [48], and does not necessarily reflect current GLP-1R expression. Validation has been previously carried out on this model in peripheral cells [16], but full validation of the central expressing neurons will require future work. The current study has achieved much towards addressing this question by employing both electrophysiological recordings and viral gene transfer to address the question of whether GLP-1R and cre-recombinase are both expressed in fully developed adult animals at the time of experimentation; in all instances tested here this was the case.

## Author contributions

ST and FR conceived the project. FR and FMG provided the transgenic mouse strains. SCC performed the immunocytochemistry and electrophysiology. JER performed the *in vivo* procedures. MKH produced the AAV virus particles. ST and SCC analysed the data, wrote the manuscript, and all authors contributed to editing and provided intellectual input.

## Figures and Tables

**Table 1:** Location of GLP-1R expressing cells in this study, compared to PPG neuron projections [1] in mouse, detected by transgene expression, rat GLP-1R expression, analysed by *in situ* hybridisation [14], and non-human primate GLP-1R expression, determined by *in situ* hybridisation [15].

Region	Mouse TG	Mouse PPG projections	Rat ISH	Monkey ISH
**Telencephalon**				
Olfactory				
Granule cell layer	++	N/A	N/A	N/A
Anterior olfactory area				
Dorsal	++	N/A	N/A	N/A
Lateral	+++	N/A	N/A	N/A
Ventral	+++	N/A	N/A	N/A
Nucleus accumbens		N/A	+^a^	++^a^
Shell	+			
Core	+			
Ventral tenia tecta	++	N/A	N/A	N/A
Claustrum	++	N/A	N/A	N/A
Lateral Septum				+^a^
Intermediate	++	N/A	++	
Dorsal	++++	N/A	N/A	
Ventral	+++	N/A	N/A	
Subfornical organ	++++	N/A	N/A	N/A
Cortex	+		-a	-a
Cingulate	+	N/A		
Piriform	++	So		
Bed nucleus of the stria terminalis	+++	+++	+	++
Septohypothalamic nuclei	++	N/A	N/A	N/A
Preoptic area				
Medial	+	N/A	N/A	N/A
Lateral	+	N/A	N/A	N/A
Ventral	++	++	N/A	N/A
Median	++	N/A	N/A	N/A
Hippocampus				+^a^
CA3				
Rostral	+	–	–	
Caudal	+++	N/A	+	
CA1 (Caudal)	++	N/A	N/A	
Dentate gyrus	++	–	N/A	N/A
Central Amygdala	++++	+	–	+++
Medial Amygdala	+++	N/A	–	++
Basolateral Amygdala	++	N/A	N/A	N/A
Cortical Amygdala	++	N/A	N/A	N/A
Organum vasculosum laminae terminalis	+	++	N/A	N/A
**Diencephalon**				
Thalamus				
Anterior thalamic nuclei	+++	N/A	–	N/A
Ventral posteriomedial thalamic nuclei	+++	N/A	N/A	N/A
Paraventricular nucleus	+	+++	N/A	++
Geniculate nucleus		N/A	N/A	N/A
Dorsolateral	++			
Pregeniculate	++			
Medial	++			
Zona incerta	+++	N/A	++	–
Nucleus reuniens	++	N/A	+	N/A
Parasubthalamic nucleus	+	N/A	N/A	N/A
Ventral anterior thalamic nucleus	+++	N/A	N/A	N/A
Posterior thalamus	+++	N/A	N/A	–
Parafascicular thalamic nucleus	++	N/A	+++	N/A
Precommissural nucleus	+++	N/A	N/A	N/A
Mediodorsal nucleus	++++	N/A	N/A	N/A
Ventromedial thalamic nucleus	++	N/A	N/A	N/A
Lateral habenular nucleus	++	N/A	N/A	N/A
Thalamic reticular formation	++	N/A	N/A	N/A
Hypothalamus				
Anterior hypothalamus	++	++	N/A	N/A
Dorsomedial hypothalamus	++	++++	+	++
Paraventricular nucleus	+++	++++	++++	+++
Arcuate nucleus	+++	+++	++++	++++
Lateral Hypothalamus	++	+++	+	++
Posterior hypothalamus	++	N/A	N/A	N/A
Suprachiasmatic nucleus	+++	N/A	N/A	N/A
Retrochiasmatic nucleus	+++	N/A	N/A	N/A
Accessory neurosecretory nuclei	++	N/A	N/A	N/A
Ventromedial hypothalamus	+++	++	+	–
**Mesencephalon**				
Periaqueductal grey	+++	+++	+++	+
Premamilliary nucleus	+	N/A	N/A	N/A
Mammilliary nucleus	++	N/A	N/A	N/A
Posterior commissure	+++	N/A	N/A	N/A
Pretectal nucleus		N/A	N/A	N/A
Anterior	++			
Medial	+++			
Posterior	+++			
Olivary	+++			
Superior colliculus	+++	N/A	++^b^	N/A
Ventral tegmental area	+	N/A	++	++
Parasubthalamic nucleus	++	N/A	N/A	N/A
**Brainstem**				
Area postrema	++++	+	++++	++++
Nucleus tractus solitarius	+	++	+++	++
Nucleus of Roller	+	N/A	N/A	N/A
Reticular formation	++	++	N/A	+
Rostral ventrolateral medulla	++	+++	N/A	N/A
Locus Coeruleus	++	+++	N/A	+

N/A – no information available; So: somata.

a Analysis of GLP-1R expression for these areas in rat/monkey is not subdivided by region.

b Analysis of GLP-1R expression for these areas in rat/monkey has been further subdivided, but not in mouse.
